# Characterization of Immune Infiltrates Associated With Radiation Necrosis in the Setting of Brain Metastases Following Stereotactic Radiosurgery and Immunotherapy: A Retrospective Cohort Analysis

**DOI:** 10.7759/cureus.43528

**Published:** 2023-08-15

**Authors:** Shirley Jiang, Robert Macaulay, Kamran Ahmed, Arnold B Etame, Hsiang-Hsuan Michael Yu

**Affiliations:** 1 Department of Radiation Oncology, Houston Lee Moffitt Cancer Center, Tampa, USA; 2 College of Medicine, University of South Florida (USF) Health, Tampa, USA; 3 Department of Pathology, Houston Lee Moffitt Cancer Center, Tampa, USA; 4 Department of Neurosurgery, Houston Lee Moffitt Cancer Center, Tampa, USA

**Keywords:** brain metastases, radiation necrosis, stereotactic radiosurgery, immunotherapy, microenvironment

## Abstract

Introduction: Radiation necrosis (RN) is caused by vascular damage and brain parenchymal injury resulting in inflammation following radiotherapy (RT) for brain metastases. The impact of immunotherapy (IO) on the immune cellular microenvironment in patients’ brain metastases is unknown. The objective of this study was to characterize the inflammatory microenvironment in the setting of RN compared to recurrent metastasis and determine whether IO treatment affects the cellular infiltrates.

Methods: Adult patients with brain metastases from solid tumors who received both systemic IO and RT prior to resection of intracranial lesions were retrospectively analyzed. The resection either showed biopsy-proven RN or recurrent tumor. A group of patients who developed RN and were not on IO was reviewed as well. A total of 18 patients were categorized into one of three groups: necrosis, IO+RT; tumor, IO+RT; and necrosis, RT. Surgical specimens were stained for immune and inflammatory components and reviewed by a neuro-pathologist who remained blinded during the analysis. The presence or absence of lymphocytes, perivascular cuffs, plasma cells, macrophages, and fibrinoid vascular changes was characterized in a semiquantitative manner.

Results: The median age was 61.5 years (range 37-82 years). Seventy-seven percent of primary cancers were melanoma. Patients with RN were more likely to exhibit immune infiltrates compared to patients with recurrent metastasis. Limited analysis showed 100% of patients in “necrosis, IO+RT” had quantifiable cell counts; conversely, 83.3% of patients in “tumor, IO+RT” lacked quantifiable cell counts. Additionally, 83.3% of patients in “necrosis, RT” showed immune cells, including lymphocytes, macrophages, plasma cells, and cuffing.

Conclusion: The immune microenvironment of brain metastasis following RT and IO showed higher levels of cell infiltrates in the RN setting versus the recurrent tumor setting. Patients who received prior IO compared to those with no IO had similar immune cell infiltrates adjacent to RN. Lower levels of immune cells in tumor recurrence following IO and RT raise the possibility that an environment lacking primed immune cells may decrease the efficacy of IO.

## Introduction

Immunotherapy (IO) represents a significant advancement and paradigm shift in the treatment of systemic cancers once refractory to conventional chemotherapy. Treatment of brain metastases has historically included surgery, stereotactic surgery, and whole-brain radiotherapy (RT). Immune checkpoint inhibition, which can be combined with RT, is becoming the emerging standard for non-small cell lung cancer and melanoma metastases, as well as other primary tumor types. Approved immunotherapies such as anti-CTLA-4 or anti-PD-1 monoclonal antibodies may enhance T-cell activation and have shown therapeutic activity against brain metastases; this effect is thought to be due to reactivating or priming the existing immune system against tumor cells. The success of these agents suggests that immune cells are present in the brain to carry out anti-tumor functions. Retrospective studies have found tumor-infiltrating lymphocytes present in over 90% of brain metastases patients [1,2]. A higher density of CD8+ T cells expressing PD-L1 was shown to correlate with an increased response to PD-1/PD-L1 blockade in various cancer types [3]. However, the presence of CD8+ T cells may not be the only factor predicting response to IO; namely, the cytotoxicity should also be considered. A 2017 study reported that during anti-PD-1 treatment of melanoma patients, the density of tumor-infiltrating lymphocytes did not significantly change, but their cytolytic activity did [4]. The mechanisms behind the efficacy of IO on tumor modulation in different patients are unclear, and there is limited information on the microenvironment of metastatic brain tumors after systemic and local treatments.

Radiation necrosis (RN) is a delayed inflammatory process and serious sequela of RT for brain metastases. The timeframe for the development of RN varies from three to 12 months after RT treatment, although occurrence years after RT has also been reported [5]. Risk factors include total dose, dose fractionation, and type of RT. Specifics about tumor biology, including receptor and mutational status, may also be associated with RN; for example, Miller et al. identified populations such as HER2-amplified breast cancers, ALK+ lung cancers, and V600 wild-type melanoma to have increased risk for RN [6]. The pathophysiology of RN entails vascular injury and perivascular inflammation, involving oligodendrocytes and astrocytes, that exacerbate blood-brain barrier disruption and associated edema [5]. Pro-inflammatory cytokines and angiogenic factors, such as vascular endothelial growth factor (VEGF), produced by microglia and astrocytes have been implicated in RN based on studies with surgical specimens [7]. The immune cell environment in the setting of RN has not been reported.

In this study, we characterized cellular infiltrates associated with RN in brain metastasis patients who received IO and RT. We are asking whether IO contributes to the inflammatory phenotype of biopsy-proven RN. Furthermore, we asked if there is a difference in the microenvironments of patients who experience tumor recurrence rather than RN and have received both IO and RT.

This article was previously presented as a meeting poster at the 2022 ASTRO Annual Meeting on October 24, 2022.

## Materials and methods

Data were reviewed retrospectively to identify patients treated by a single surgeon at our institution from 2013 to 2021. Twelve patients received IO and RT before resection of the treated metastasis as part of routine clinical care. Pathological analysis of the tissue samples revealed six patients with RN at the surgical site and six patients with tumor recurrence at the surgical site. Another separate group of six patients who only received RT without IO and underwent resection with pathologically confirmed RN (no viable tumor) was included. The 18 total patients were categorized into three groups: necrosis, IO+RT; tumor, IO+RT; and necrosis, RT. The group size and case selection were limited by the specific inclusion criteria of preoperative IO and/or RT, RT to the same site as the resection site, and confirmation of RN or tumor recurrence to the same resection site. Clinical data collected include age at surgery, tumor location, IO start and end dates, type and dose of RT, other prior radiation, and steroids before surgery in addition to other clinical parameters.

All patients had RT either as stereotactic radiosurgery (SRS) or fractionated stereotactic RT (FSRT). IO included ipilimumab, pembrolizumab, and/or nivolumab. Surgical specimens were stained for routine immune and inflammatory components, and reviewed by a neuro-pathologist who remained blinded during the analysis. Immune infiltrates if present were characterized. The study was approved by the Moffitt Cancer Center Institutional Review Board (protocol number 17324).

Stereotactic radiation technique

Brain metastases were assessed using MRI (Siemens Sonata, Siemens Medical Systems, Erlangen, Germany) with one-millimeter slices for treatment planning purposes prior to the delivery of radiation. The MRI image was co-registered and fused with CT imaging (General Electric Medical System, Milwaukee, WI). A uniform one-millimeter expansion of the gross tumor volume was used to create the planning target volume (PTV). All brain metastases were treated with SRS in a single session except four, which were treated with FSRT. Four metastases were in the post-operative setting. Doses were prescribed to ensure coverage of at least 95% of the PTV with the prescription dose. Treatments were delivered using multiple dynamic conformal arcs or intensity-modulated RT. Patient immobilization was achieved by using a commercially available head mask fixation system (BrainlabAG, Feldkirchen, Germany). Treatments were delivered with the BrainLab Novalis Classic or Varian Truebeam with six MV photons with image guidance using ExacTract positioning system.

Pathology assessment and scoring

Tissue samples were formalin-fixed and paraffin-embedded. Samples were variously immunostained with MelanA, HMB45, MITF, GFAP, CAM5.2, CK7, CK20, TTF1, GATA3, ThyGl, CD68, CD3, CD20, and the proliferation marker Ki-67, depending on the underlying tumor type. Immune and inflammatory components quantified were lymphocytes, perivascular cuffs, plasma cells, macrophages, and fibrinoid vascular changes. Semiquantitative evaluation criteria to judge cellular infiltrates included the following levels: absent (zero), sparse or mild (one), and moderate or intense (two).

Statistical analysis

Comparisons between groups were made using Fisher’s exact analysis. Descriptive statistics were used to summarize the cohort including median and range for continuous variables or counts and percentages for categorical variables. A two-tailed p<0.05 was considered statistically significant.

## Results

Table 1 shows the demographic and tumor characteristics of all 18 patients that met the inclusion criteria. The median age was 61.5 years (range 37-82 years). About 77.7% of primary cancers were melanoma, and other cancers included non-small cell lung cancer (5.6%), breast (5.6%), thyroid (5.6%), and nasopharyngeal squamous cell carcinoma (5.6%). Patients were all treated with SRS or FSRT with doses ranging from 13 to 32.5 Gy.

**Table 1:** Patient and treatment characteristics IO: immunotherapy, RT: radiotherapy, NSCLC: non-small cell lung cancer, SCC: squamous cell carcinoma, WBRT: whole brain radiotherapy

Characteristic	Necrosis, IO+RT (n=6)	Tumor, IO+RT (n=6)	Necrosis, RT (n=6)	All patients (n=18)
Median age at surgery (years)	72	52.5	61	61.5
Male	4	3	3	10
Female	2	3	3	8
Tumor location				
Frontal	4	4	2	10
Temporal	1	1	1	3
Occipital	1	0	0	1
Cerebellar	0	0	1	1
Parietal	0	1	2	3
Primary cancer				
Melanoma	6	6	2	14
NSCLC	0	0	1	1
Breast	0	0	1	1
Thyroid	0	0	1	1
Nasopharyngeal SCC	0	0	1	1
Melanoma BRAF +	1	2	1	4
Melanoma BRAF wildtype	5	4	1	10
Radiotherapy				
Single fraction	4	4	3	11
Multi-fraction	1	1	2	4
Mean radiation dose (Gy)	20.5	22.2	23.3	22
Prior WBRT	1	2	0	3
Prior to additional local radiotherapy	0	3	2	5

The comparison of the distribution of microenvironment cell counts between “necrosis, IO+RT” and “rumor, IO+RT” is reported in Table 2. The presence or absence of individual immune cell types is compared between the two groups. In general, more patients in the “necrosis, IO+RT” group had immune cells present on surgical specimens than those in the “tumor, IO+RT.” In particular, 83.3% of “necrosis, IO+RT” patients had detectable lymphocytes versus 16.7% of “tumor, IO+RT” patients (p=0.0810). Macrophages and fibrinoid vascularity were present in 66% of “necrosis, IO+RT” patients and 0% of “tumor, IO+RT” patients (p=0.0606). Given the small number of patients for each group and no significant difference in individual cell type analysis, the semi-quantified cell types were combined for further analysis. The combination of semiquantitative scores for lymphocytes, plasma cells, macrophages, and cuffing (each cell type scoring either zero, one, or two) revealed 100% of “necrosis, IO+RT” patients having a combined semiquantitative score greater than zero: 33% of patients scored a total of one, 33% scored a total of two, and 33% scored a total of three. In contrast, 83.3% of “tumor, IO+RT” patients scored a total of zero, and 16.7% scored a total of two (p=0.01515). Looking at the presence or absence of any cell type, 100% of “necrosis, IO+RT” patients had observed immune cells compared to 16.7% of “tumor, IO+RT” patients (p=0.01515).

**Table 2:** Frequency distribution of individual and combined cell types (necrosis, IO+RT compared with tumor, IO+RT) IO: immunotherapy, RT: radiotherapy, * indicates p<0.05. Values represent the percentage of patients within each comparison group with the presence or absence of various cell types

Individual cell type		Necrosis, IO+RT (%)	Tumor, IO+RT (%)	p-value
Lymphocytes	Present	83.3	16.6	0.0801
	Absent	16.6	83.3	
Cuffing	Present	50	0	0.1818
	Absent	50	100	
Plasma cells	Present	0	16.6	1
	Absent	100	83.3	
Macrophages	Present	66.6	0	0.0606
	Absent	33.3	100	
Fibrinoid	Present	66.6	0	0.0606
	Absent	33.3	100	
Combined cell types				
Semiquantitative score	0	0	83.3	0.01515*
	1	33.3	0	
	2	33.3	16.6	
	3	33.3	0	
Dichotomous score	Present	100	16.6	0.01515*
	Absent	0	83.3

The comparison of microenvironment cell counts between “necrosis, IO+RT” and “necrosis, RT” groups was more similar, as seen in Table 3. In all these patients with RN, both groups had identical ratios of patients with the following individual cell types: lymphocytes (83.3% present, 16% absent), cuffing (50% present, 50% absent), and macrophages (66.7% present, 33.3% absent). For the combined cell type analysis, 100% of “necrosis, IO+RT” patients had a combined score greater than zero, and 83.3% of “necrosis, RT” patients had a combined score greater than zero.

**Table 3:** Frequency distribution of individual and combined cell types (necrosis, IO+RT compared with necrosis, RT) IO: immunotherapy, RT: radiotherapy. Values represent the percentage of patients within each comparison group with the presence or absence of various cell types

Individual cell type		Necrosis, IO+RT (%)	Necrosis, RT (%)	p-value
Lymphocytes	Present	83.3	83.3	1
	Absent	16.6	16.6	
Cuffing	Present	50	50	1
	Absent	50	50	
Plasma cells	Present	0	50	0.1818
	Absent	100	50	
Macrophages	Present	66.6	66.6	1
	Absent	33.3	33.3	
Fibrinoid	Present	66.6	100	0.4545
	Absent	33.3	0	
Combined cell types				
Semiquantitative score	0	0	16.6	0.6104
	1	33.3	16.6	
	2	33.3	0	
	3	33.3	50	
	4	0	16.6	
Dichotomous score	Present	100	83.3	1
	Absent	0	16.6	

## Discussion

In this initial exploratory analysis and characterization of cellular immune infiltrates in brain metastasis patients receiving radiotherapy and immunotherapy, we examined the microenvironment signature of RN and tumor recurrence in patients with similar treatment backgrounds (receiving both radiotherapy and immunotherapy prior to resection). The comparison of individual immune cells demonstrated a trend toward higher levels of infiltrates in patients with RN compared to those with tumor recurrence, especially for lymphocytes, macrophages, and fibrinoids. The combined scores for all cell types showed 100% of RN specimens having present immune cells of any type compared to 16.6% of recurrent tumor specimens.

Studies of RN after the utilization of radiotherapy and immunotherapy are limited in the literature, with most as retrospective studies. The rationale for combining both types of therapies includes radiotherapy potentially inducing the release of inflammatory cytokines and tumor antigens and inhibiting immune suppressive cells, thereby allowing immunotherapy to have an enhanced effect [8]. PD-L1 may also be upregulated in the tumor microenvironment following radiotherapy, suggesting a synergistic response between radiotherapy and immunotherapy [9]. Preliminary studies have found varying rates of RN in patients with immunotherapy, possibly from different definitions used for “treatment-related imaging changes.” Weingarten et al. found the combination to have a mild risk of developing symptomatic RN (incidence of 7%) compared to SRS alone [10]. A 2021 meta-analysis included 44 studies of melanoma brain metastases patients to address combinatorial radiotherapy + immunotherapy on medial overall survival and CNS toxicity, compared with either alone. It reported better survival outcomes for the radiotherapy + immunotherapy arm compared to either radiotherapy (HR: 0.595 (0.489-0.723, p <.001)) or immunotherapy (HR: 0.693 (0.526-0.913, p = .001)) arm alone, along with a nonsignificant increase in Grade 3 or higher RN [11].

The role of systemic immune cells in relation to the formation or treatment of brain metastases has yet to be defined. Investigations of tumor-infiltrating lymphocytes in melanoma brain metastases by Berghoff et al. found prominent lymphocytic infiltrates in the microenvironment of the brain, associated with high PD-L1 expression as well [12]. This initial characterization of immune cells in brain metastases may underlie the efficacy of anti-PD-1 immunotherapy (such as pembrolizumab and nivolumab) in melanoma patients particularly. Furthermore, treatment for metastatic melanoma using adoptive cell therapy with tumor-infiltrating lymphocytes resulted in a high rate of tumor regression in patients who were lymphodepleted [13]. In breast cancer, which is viewed as a lower immunogenic cancer, the presence of tumor-infiltrating lymphocytes showed prognostic value with higher response rates to neoadjuvant chemotherapy [14]. These findings imply the importance of the cell types present in the tumor microenvironment on treatment outcomes. Our characterization shows that the more prevalent cell types in RN include lymphocytes and macrophages.

The effect of prior immunotherapy treatment was also analyzed in this study by comparing “necrosis, IO+RT” and “necrosis, RT” groups. We found that levels of individual cell types in resection specimens were similar between these two groups. No significant difference was found between the combined cell types scores as well. Our results did not suggest a relationship between immunotherapy and the cellular microenvironment in the setting of RN and, thus, did not show a difference in inflammatory phenotype in RN patients who received immunotherapy compared to those who did not. A report by Colaco et al. reported an increase in the incidence of RN with the use of immunotherapy, compared to chemotherapy or targeted therapy [15]. The mechanism is unknown but may involve exacerbating the inflammatory response which could be examined histologically.

Of note, we noticed a feature in the semiquantitative scores of “necrosis, RT” patients who seem to have a higher intensity of cells. For example, 66.6% of patients in this group scored a "two" in the semiquantitative evaluation of at least one cell type. This is in contrast to 16.6% of patients in “necrosis, IO+RT” and 0% of patients in “tumor, IO+RT.” This finding may be due to the evolutionary change of RN on brain parenchyma, a process thought to involve endothelial damage and vascular permeability that may allow the extravasation of inflammatory cells. An early response in RN and the role of the inflammatory response has shown lymphocytic infiltration as well as VEGF produced from astrocytes [7].

Our study has several limitations, including the retrospective nature that does not allow uniform patient characteristics and therapy protocols. In addition, data on the cell immune infiltrates for our study was collected from routine pathologic evaluation; further testing to quantify detailed immune cell types was not performed as it is not routinely performed for clinical practice. Additionally, our small sample size only allowed for limited analysis of the group comparisons statistically. A nonsignificant trend toward differences between “necrosis, IO+RT” and “tumor, IO+RT” was found, but a small sample size limits further characterization. The majority composition of melanoma as primary cancer in these two groups may limit the external generalizability as it does not represent all brain metastases tumor behaviors. It would be ideal to have the cellular density of brain metastases at multiple time points for comparison within each patient, including before any treatment. Nevertheless, our investigation provides a cross-sectional view of brain metastases patients with various treatment combinations and outcomes.

## Conclusions

In conclusion, our analysis showed that the immune microenvironment of brain metastasis following radiotherapy and immunotherapy shows higher levels of cell infiltrates in the RN setting versus the recurrent tumor setting. A lower level of presence of immune cells in tumor recurrence following immunotherapy + radiotherapy may suggest an environment lacking primed immune cells with the implication of potential decreased efficacy in immunotherapy. We did not detect a difference in inflammatory phenotype in RN patients who received immunotherapy compared to those who did not. Our findings affirm our initial hypothesis that there are distinct histopathologic characteristics of the tumor environment in the setting of RN. Further studies are needed to confirm our findings in a larger patient cohort and determine if patterns of immune cell infiltration may differ by tumor histology.
